# Effects of CO_2_ laser irradiation on matrix-rich biofilm development formation–an in vitro study

**DOI:** 10.7717/peerj.2458

**Published:** 2016-11-01

**Authors:** Bruna Raquel Zancopé, Vanessa B. Dainezi, Marinês Nobre-dos-Santos, Sillas Duarte, Vanessa Pardi, Ramiro M. Murata

**Affiliations:** 1 Department of Pediatric Dentistry, Piracicaba Dental School, University of Campinas-UNICAMP, Piracicaba, São Paulo, Brazil; 2 Division of Restorative Sciences, Ostrow School of Dentistry of University of Southern California, Los Angeles, California, USA; 3 Division of Periodontology, Diagnostic Sciences and Dental Hygiene, Ostrow School of Dentistry of University of Southern California, Los Angeles, California, USA; 4 Department of Foundational Sciences, School of Dental Medicine, East Carolina University, Greenville, North Carolina, USA

**Keywords:** Lasers, Biofilm, Caries, Prevention & control

## Abstract

**Background:**

A carbon dioxide (CO_2_) laser has been used to morphologically and chemically modify the dental enamel surface as well as to make it more resistant to demineralization. Despite a variety of experiments demonstrating the inhibitory effect of a CO_2_ laser in reduce enamel demineralization, little is known about the effect of surface irradiated on bacterial growth. Thus, this in vitro study was preformed to evaluate the biofilm formation on enamel previously irradiated with a CO_2_ laser (λ = 10.6 µM).

**Methods:**

For this in vitro study, 96 specimens of bovine enamel were employed, which were divided into two groups (n = 48): 1) Control-non-irradiated surface and 2) Irradiated enamel surface. Biofilms were grown on the enamel specimens by one, three and five days under intermittent cariogenic condition in the irradiated and non-irradiated surface. In each assessment time, the biofilm were evaluated by dry weigh, counting the number of viable colonies and, in fifth day, were evaluated by polysaccharides analysis, quantitative real time Polymerase Chain Reaction (PCR) as well as by contact angle. In addition, the morphology of biofilms was characterized by fluorescence microscopy and field emission scanning electron microscopy (FESEM). Initially, the assumptions of equal variances and normal distribution of errors were conferred and the results are analyzed statistically by t-test and Mann Whitney test.

**Results:**

The mean of log CFU/mL obtained for the one-day biofilm evaluation showed that there is statistical difference between the experimental groups. When biofilms were exposed to the CO_2_ laser, CFU/mL and CFU/dry weight in three day was reduced significantly compared with control group. The difference in the genes expression (Glucosyltransferases (gtfB) and Glucan-binding protein (gbpB)) and polysaccharides was not statically significant. Contact angle was increased relative to control when the surface was irradiated with the CO_2_ laser. Similar morphology was also visible with both treatments; however, the irradiated group revealed evidence of melting and fusion in the specimens.

**Conclusion:**

In conclusion, CO_2_ laser irradiation modifies the energy surface and disrupts the initial biofilm formation.

## Introduction

Dental caries, a biofilm-related disease, remains among the most prevalent human infections disease affecting both children and adults worldwide (Do, 2012; Dye et al., 2008; Marcenes et al., 2013). Despite its decline over the last decades primarily due to the widespread use of fluoride compounds, caries disease activity in children is as high as 60–70% (Murata & Pardi, 2007; Taubman & Nash, 2006; García-Godoy & Hicks, 2008; Greene, 2005; Hausen, 1997; Peterson, 2003). Colonization of tooth surfaces by *mutans streptococci* and its interaction with constituents from the host’s diet is associated with the etiology and pathogenesis of dental caries in humans (Bowen, 2002; Marsh, 2003). Although dental biofilms are composed of diverse and complex oral microorganisms, *Streptococcus mutans* is considered the primary etiologic agent of dental caries, which has an important role in the initiation and progression of the dental caries (Loesche, 1986; Wang et al., 2013), as it uses carbohydrates, such sucrose, to synthesize extracellular polysaccharides and can survive under low pH conditions, leading to enamel demineralization (Rölla, 1989).

Laser therapy has been studied as a promising alternative in the prevention of caries. Different types of lasers such as Nd:YAG, Argon, Er:YAG and carbon dioxide (CO_2_) have been studied for their potential use in dentistry. The use of high-power lasers has been suggested as the treatment of tooth enamel in order to obtain more resistant surfaces to acids produced by cariogenic bacteria (Featherstone et al., 1991; Featherstone et al., 1998; Hsu et al., 2000; Kantorowitz, Featherstone & Fried, 1998). A study conducted by Armengol et al. (2003) showed that morphological changes on enamel and dentin were greater when Er:YAG laser and Nd:YAP laser were employed, which was asscociated with a greater free surface energy. Intriguingly, Venault et al. (2014) reported that the coating of hydroxyapatite with a polyethyleneimine (PEI) polymer inhibited the adsorption and showed a 70% inhibition of oral bacterial adhesion on human teeth. However, the applicability of superhydrophobic and superhydrophilic surfaces in the dental field remains to be investigated. The conventional wisdom is that a reduction of surface roughness and surface free energy of a dental material coincides with a decrease in microbial adherence and proliferation (Buergers et al., 2009; Teughels et al., 2006). These results suggest that surface parameters such as the chemical composition and topography might be key parameters for optimizing the enamel surface properties in order to reduce biofilm formation on their surfaces.

The CO_2_ laser acts on enamel demineralization to reduce the acid solubility. Previous studies have also shown significant inhibition of enamel demineralization following treatment with a CO_2_ laser (Hsu et al., 2001; Nobre-dos-Santos, Featherstone & Fried, 2001; Rodrigues, dos Santos & Featherstone, 2006; Steiner-Oliveira et al., 2006; Tagliaferro et al., 2007). There are several hypotheses that attempt to explain the mechanisms by which a CO_2_ laser inhibits tooth enamel demineralization. One possible explanation is based on reducing enamel solubility caused by the melting and recrystallization of hydroxyapatite crystals (Nelson et al., 1986). However, there is no report in scientific literature showing whether these morphological alterations promoted by laser irradiation could change the energy surface and consequently to modify the development of biofilm enamel surface. Thereby, the aim of this study was to evaluate the biofilm formation on enamel previously irradiated with a CO_2_ laser (λ = 10.6 μM).

## Material and Methods

### Experimental design

**Figure 1:**
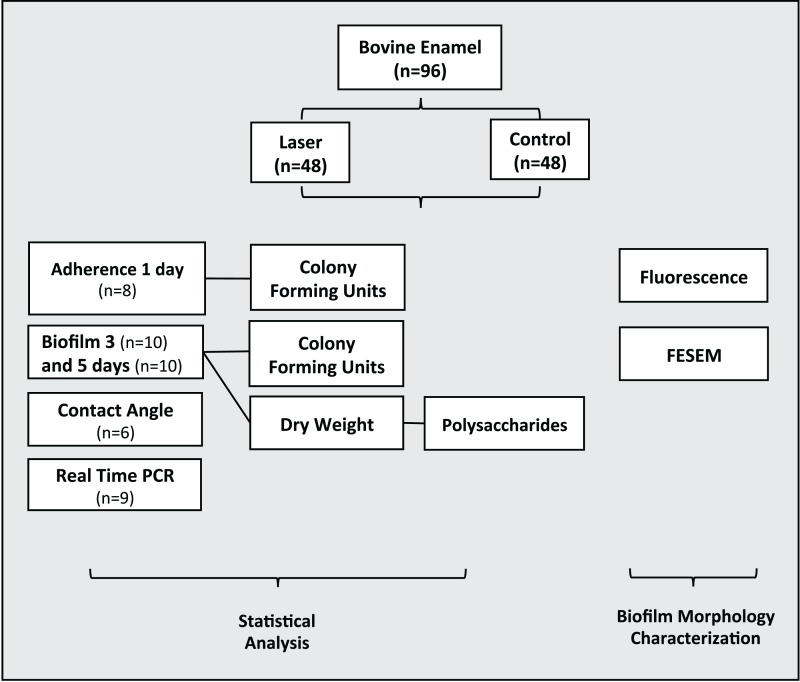
Flowchart of the experimental design of the study.

Ninety-six dental enamel specimens were previously prepared were randomly allocated in two groups (n = 48): 1) Control-non-irradiated surface and 2) Irradiated enamel surface. Biofilms were grown on the enamel specimens by one, three and five days under intermittent cariogenic condition in the irradiated and non-irradiated surface. The following analyses were performed: adherence test with one-day biofilm formation (n = 8), bacterial viability, colony forming units–CFU/mg of biofilm dry weight, dry weight and polysaccharides analysis with three-day (n = 10) and five-day (n = 10) biofilm formation. Real time Polymerase Chain Reaction (PCR) (n = 9) and Contact angle (n = 6). Morphological surface changes of three specimens of each group were examined by Field Emission Scanning Electron Microscopy (FESEM) and by fluorescence microscopy (Fig. 1).

### Tooth selection and sample preparation, experimental model

To perform this in vitro study, 96 sound bovine incisors that were free from caries, macroscopic cracks, abrasions as well as staining assessed by visual examination, were stored in a 0.1% thymol solution, and sectioned mesiodistally using a water-cooled diamond saw in a cutting machine (Isomet; Buehler, Lake Bluff, IL, USA). The tooth halves were polished for 30 s using a 5 μM alumina/water suspension micropolish (Instrumental, Jabaquara, SP, Brazil) to expose fresh enamel. The specimens were coated with an acid-resistant varnish leaving a window of 4 mm^2^ of exposed enamel in the middle of the surface. The teeth were sterilized using oxide ethylene (Acecil Central Esterilizacao com Ind. Ltda de Campinas-SP, Campinas, Brasil).

### Laser irradiation parameters

For this study, we based the irradiation parameters in a previous work by our group (Steiner-Oliveira et al., 2006) showing that 11.3 J/cm^2^ was able to produce chemical and morphological changes that could reduce the acid reactivity of enamel without compromising pulp vitality. To perform enamel surface irradiation, a pulsed CO_2_ laser at 10.6 μM wavelength (Union Medical Engineering Co. Model UM-L30; Yangju-si, Gyeonggi-Do, Korea) was used with the following parameters: 10-ms pulse duration, 10-ms of time off, 50-Hz repetition rate, beam diameter of 0.3-mm (according to laser manufacturer), single pulse fluence of 11.3 J/cm^2^ and total fluence delivered to treated area of 300 J/cm^2^. The average power output was measured at 0.4 W using a power meter (Scientech 373 Model-37-3002; Scientech Inc., Boulder, CO, USA). To provide uniform coverage of enamel surface (4 mm^2^), we used a X-Y positioning platform at a 10-mm distance from the tip of the handpiece to the enamel surface. The handpiece was positioned perpendicularly to the enamel surface, and we irradiated the samples once in each direction, slowly by manually moving the X-Y positioning platform horizontally and vertically, in order to promote homogeneous irradiation of the entire specimen experimental surface area.

### Biofilm formation and analysis

*Streptococcus mutans* UA159 (ATCC 700610), a virulent cariogenic pathogen, was used for the biofilm study. Biofilms were grown in Brain Heart Infusion (BHI) broth containing 1% (w/v) sucrose and were kept undisturbed for 24 h to allow initial biofilm formation. Medium was replaced twice daily. Biofilms of *S. mutans* UA159 were formed on specimens of bovine enamel placed in 2 mL of medium containing 1% sucrose, in 24-well cell culture plates, at 37 °C, 5% CO_2_, for three and five days, which were dip-washed three times with Phosphate Buffered Saline (PBS) at the end of each experimental period. The biofilms were removed using a metallic spatula, immersed in a falcon tube with PBS and subjected to sonication using three 15 s pulses at an output of 7 W (Fisher Scientific, Sonic Dismembrator model 100; NH, USA). The suspension was used as previously described (Duarte et al., 2006) for dry weight, bacterial viability (colony forming units—CFU/mg of biofilm dry weight), and polysaccharide analyses (EPS-soluble, EPS-insoluble and intracellular polysaccharides—IPS) (Duarte et al., 2011).

*S. mutans* adherence test was performed in day 1 of biofilm formation. After that, the numbers of colonies were counted and the value of log CFU/mL was calculated (Branco-de-Almeida et al., 2011).

### Dry weight and bacterial viability

Three volumes containing cold ethanol (−20 °C) were added to 1 mL biofilm suspension, and the resulting precipitate was centrifuged (10,000 g for 10 min at 4 °C). The supernatant was discarded, and the pellet was washed with cold ethanol, and then lyophilized and weighed (Duarte et al., 2006).

An aliquot (0.1 mL) of the homogenized suspension was serially diluted (1:10, 1:100, 1:1,000, 1:10,000, 1:100,000, 1:1,000,000) and plated on blood agar. The plates were incubated in 5% CO_2_ at 37 °C for 48 h, and the number of CFU mg-1 of biofilm dry weight were determined (Murata et al., 2010).

### Polysaccharide analysis

Soluble and insoluble extracellular polysaccharides (EPS-soluble and EPS-insoluble) were analyzed as previously described (Duarte et al., 2006). The polysaccharide content was expressed per mg of polysaccharide by dry weight of total biofilm. Briefly, an aliquot (2 mL) of the suspension was sonicated for 30 s pulses at an output of 7 W and centrifuged at 10,000 g for 10 min at 4 °C. The supernatant was collected and the biofilm pellet was resuspended and washed in 5 mL of milli-Q water. This procedure was repeated three times. The supernatant was used for the EPS-soluble assay and biofilm pellet was used for the EPS-insoluble assay. All of the supernatants were pooled and three volumes of cold ethanol were added, and the resulting precipitate was collected by centrifugation and resuspended in 5 mL Milli-Q water; the total amount of carbohydrate was determined by the phenol—sulfuric acid method (Dubois et al., 1951). The EPS-insoluble was extracted using 1 N NaOH (1 mg biofilm dry weight/0.3 mL of 1 N NaOH) under agitation for 1 h at 37 °C. The supernatant was collected by centrifugation, and the precipitate was resuspended again in 1N NaOH; this procedure was repeated three times. The total amount of carbohydrate was determined by colorimetric method with phenol sulfuric acid (Dubois et al., 1951).

### Quantitative real-time PCR

All RNA was isolated from biofilm (three days). The *S. Mutans* RNA were isolated and purified by using the Ribopure Kit (Life Technology, Grand Island, NY, USA). A NanoPhotometer P360 (Implen, Westlake Village, CA, USA) was used to quantify the total RNA extracted. Reverse transcription of the RNA into cDNA was carried out by using iScript Advanced cDNA synthesis Kit for RT-qPCR (Biorad, Hercules, CA, USA) according to the manufacturer’s instructions. Real-time PCR was conducted by using iQ SYBR Green Supermix (Biorad, Hercules, CA, USA) (Klein et al., 2010). The *S. Mutans* primers for the genes: Glucosyltransferase (gtfB), Glucan-binding protein (gbp), at 10 μM were used. The standard curves were used to transform the critical threshold cycle (Ct) values to the relative number of cDNA molecules. Relative expression was calculated by normalizing each gene of interest to the *S. mutans* 16S rRNA gene, which is a well-established reference gene (Table 1). PCR amplification was performed by using 20 μL reaction mix per well in a 96 well plate. The reactions were conducted at 95 °C for 3 min, followed by 40 cycles of 15 s at 95 °C and 1 min at 60 °C. After PCR, the melting curve was obtained by incubating the samples at increasing increments of 0.5 °C from 55 to 95 °C (Klein et al., 2009; Koo et al., 2006).

**Table 1:** Primers used for RT-qPCR.

GenBank locus tag	Gene name	Primer sequence (forward and reverse)
	16S rRNA	ACCAGAAAGGGACGGCTAAC
		TAGCCTTTTACTCCAGACTTTCCTG
SMU.1004	gtfB	AAACAACCGAAGCTGATAC
		CAATTTCTTTTACATTGGGAAG
SMU.22	gbpB	ATACGATTCAAGGACAAGTAAG
		TGACCCAAAGTAGCAGAC

### Contact angle–wettability measurement

Wettability of enamel after treatments was evaluated by contact angle measurements. The sessile drop method was performed using Digidrop GBX goniometer (Labometric Lda, Leiria, Portugal) with enamel surface (control and laser). Briefly, deionized water was loaded into a 3 mL syringe (Luer-Lok™ Tip; BD, Franklin Lakes, NJ, USA) and coupled to the goniometer. Droplets (≅ 1 μL) were careful applied on the different enamel surfaces using a 22-gauge needle (Injex Ltda, São Paulo, SP, Brazil). Ten drops of water were dispensed on the enamel surface. The measurement of contact angle was accomplished immediately after the water drop has formed on enamel surface. The test was accomplished at room temperature and the drop images captured without external lights interferences. Images were frozen by PixeLink system (Barrington, IL, USA) and the measurements were made by the GBX Digidrop Windrop software (GBX Instruments, Bourg de Péage, France). The focus of camera used to capture the images was adjusted in relation to the position of the table with glass slide surface and the needle tip. The right and left angles were measured in degrees of the contact angle and average automatically calculated by GBX Digidrop software (GBX Instruments, Bourg de Péage, France). The average obtained from each specimen and from each group was submitted to statistical analysis (Paris et al., 2007).

### Field emission scanning electron microscopy

This analysis aimed to evaluate the surface of specimens after CO_2_ LASER irradiation and biofilm formation. All specimens were first mounted on aluminum stubs and sputter-coated with gold (∼10–12 nm thickness) using a BAL-TEC SCD 050 sputter coater (Wetzlar, Liechtenstein/Vienna, Austria). Observations were made with a JEOL JSM-7001 Field Emission Scanning Electron Microscope (Jeol, Peabody, MA, USA) operating at 15 kV and using magnifications up to 2500X (Weber et al., 2014).

### Fluorescence microscopy

The distribution of dead and live *S. mutans* was examined after 1, 3 and 5 days of biofilm using the Viability/Cytotoxicity Assay Kit LIVE/DEAD® BacLight™ Bacterial Viability (Life Technologies, Carlsbad, CA, USA) for microscopy which contains a The LIVE/DEAD BacLight Bacterial Viability Kits employ two nucleic acid stains—the green-fluorescent SYTO® 9 stain and the red-fluorescent propidium iodide stain. These stains differ in their ability to penetrate healthy bacterial cells. When used alone, SYTO 9 stain labels both live and dead bacteria. In contrast, propidium iodide penetrates only bacteria with damaged membranes, reducing SYTO 9 fluorescence when both dyes are present. Thus, live bacteria with intact green membranes fluoresce, while dead bacteria with damaged membranes fluoresce red were evidenced. Fluorescent images of the double staining were captured using fluorescence microscopy (EVOS fl microscope AMG; Bothell, WA, USA) (Rolland et al., 2006).

### Statistical analysis

The Lilliefors test showed that data of CFU, CFU/dry weight on day 3, dry weight on day 5 and insoluble polysaccharide did not follow normal distribution and were analyzed by Mann-Whitney test. Results of adherence test on day 1, dry weight on day 3, CFU, CFU/dry weight on day 5, soluble polysaccharides, real time PCR and contact angle did follow normal distribution and were analyzed by T-test, Data normality and the other analyses were performed using BioEstat 5.0 (Mamirauá, Belém, PA, Brazil) with a 5% significance level.

To evaluate the surface of specimens after treatments and biofilm formation and distribution of dead and live *S. mutans* after one, three and five days of biofilm formation, field emission scanning electron and fluorescence microscopy were respectively performed for illustration.

## Results

Table 2 showed, the results of three and five days of biofilm formation. The results on day 3 showed that the values of CFU/mL for irradiated group were significantly (p < 0.05) less when compared to the control group. The normalized data (CFU/dry weight) showed noteworthy reduction (p < 0.05) by the irradiation on day 3. However, no statistical difference was found between the groups in day 3 for dry weight values (p > 0.05). There were no statistical difference found between the groups in CFU/mL, dry weight and CFU/dry weight on day 5 (p > 0.05).

**Table 2:** The content of CFU/mL, dry weight (mg/mL), CFU/dry weight in *S. mutans* biofilm. Data represent the mean values and standard deviations.

Groups	Biofilm					
	Day 3			Day 5		
	CFU/mL	Dry weight (mg/mL)	CFU/dry weight	CFU/mL	Dry weight (mg/mL)	CFU/dry weight
Control	8.60 ± 0.33^a^	6.55 ± 0.37^a^	7.80 ± 0.31^a^	7.72 ± 0.29^a^	10.41 ± 0.92^a^	6.70 ± 0.29^a^
Laser	7.78 ± 0.16^b^	6.70 ± 0.64^a^	6.99 ± 0.08^b^	7.67 ± 0.29^a^	11.38 ± 5.72^a^	4.31 ± 1.16^a^

**Note:**

Means followed by distinct letters (a, b) are statistically different by T-test and Mann-Whitney test. (p < 0.05).

**Figure 2:**
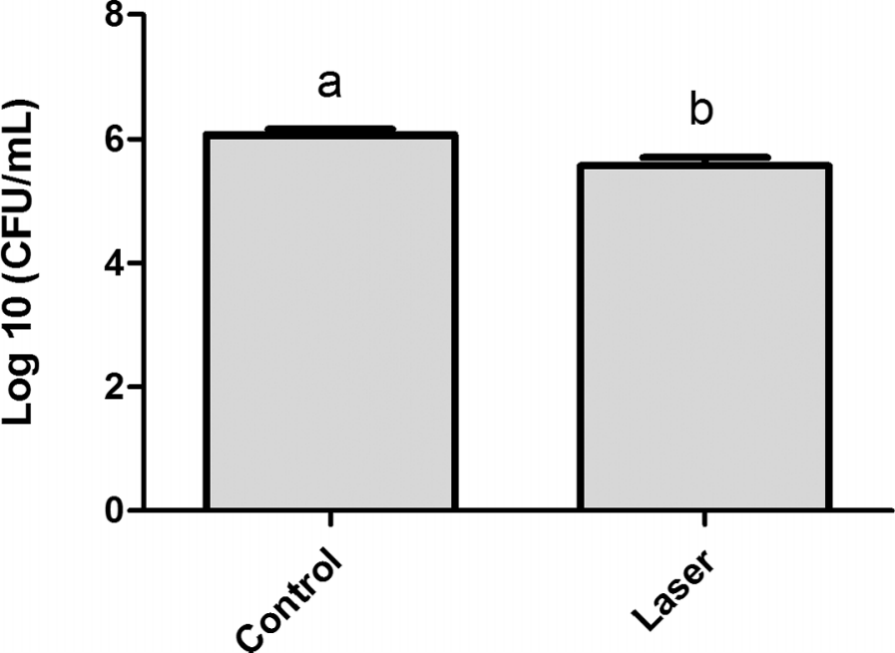
Streptococcus mutans adherence test performed in day 1 of biofilm (expressed in log CFU/mL). Values marked by the distinct letters are significantly different from each other. T-test (p < 0.05).

The results obtained for the analysis of *S. mutans* adherence to enamel surface after laser irradiation and biofilm formation are presented in Fig. 2. The values of log CFU/mL obtained for the one-day biofilm evaluation showed that although the difference between the means had been small (control group Log = 6.06 ± 0.23, irradiated group Log = 5.56 ± 0.35) there is statistical difference between the two groups (p < 0.05).

Table 3 showed that soluble and insoluble polysaccharides (μg PSA/mg dry weight) were unaffected by enamel irradiation (p > 0.05).

**Table 3:** The content of EPS-soluble, EPS-insoluble in *S. mutans* biofilm (expressed in μg/mg of biofilm). Data represent the mean values and standard deviations.

Groups	Polysaccharides (μg PSA/mg dry weight)			
	Soluble		Insoluble	
	Day 3	Day 5	Day 3	Day 5
Control	4.92 ± 1.51^a^	4.89 ± 2.13^a^	7.20 ± 1.33^a^	8.84 ± 2.80^a^
Laser	4.32 ± 1.29^a^	4.31 ± 1.27^a^	8.20 ± 2.92^a^	8.93 ± 1.31^a^

**Note:**

Values marked by the different letters (a) are significantly different from each other (p > 0.05). T-test was employed for soluble polysaccharide and Mann Whitney test for insoluble polysaccharide.

Contact Angle–Wettability Measurement analysis results after enamel treatment were shown in Table 4. This result showed that contact angle was higher for the irradiated group (87.6 ± 9.41) than for the control group (76.0 ± 3.33) and the difference between the two groups was statistically significant (p < 0.05).

**Table 4:** The content of contact angle. mutans biofilm. Data represent the mean values of angle (°) and standard deviations.

Groups	Contact angle (°)
Control	76.0 ± 3.33^a^
Laser	87.6 ± 9.41^b^

**Note:**

Values marked by the different letters (a, b) are significantly different from each other. T-test (p < 0.05).

**Figure 3:**
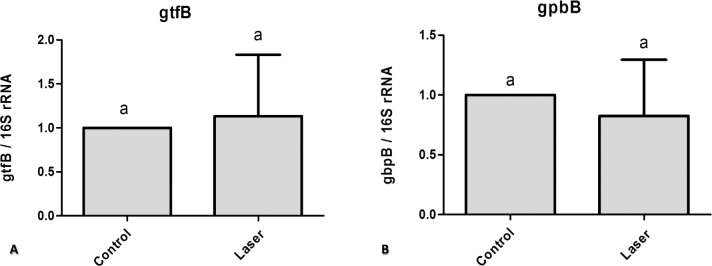
Real time quantitative information about gene expression in *S. mutans* biofilm after treatments with/without laser irradiation on enamel surface. (A) gtfB (B) gbpB. Values marked by the same letters are not significantly different from each other (p > 0.05). T-test (p > 0.05).

To assess the effect of CO_2_ laser irradiated surface on *S. mutans* gene expression we used quantitative real time PCR. We compared control non-irradiated samples to irradiated samples. The difference in the genes expression (gtfB and gbpB) was not statically significant (Fig. 3).

**Figure 4:**
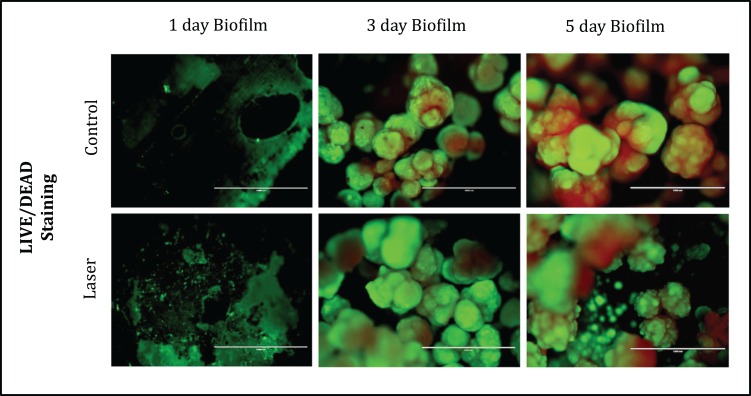
Fluorescence Microscopy showing representative images of bacteria in biofilms after 1, 3 and 5 days of biofilm. Multidimensional imaging of live (green) and dead (red) bacteria.

Fluorescence Microscopy representative images of bacteria in biofilms after one, three and five days of biofilm were shown in Fig. 4. Multidimensional imaging of live (Green) and dead (red) bacteria can be observed at different times of *S. mutans* biofilm. Similar results were found in both groups of treatment. Biofilms formed on specimens became denser from day 1 to day 5. The image on day 1 showed primarily few amounts of live bacteria, with no dead cells. In contrast, substantial increases in dead bacteria occurred with the increase of the days. In the last day (day 5), biofilms consisted of primarily dead bacteria, connected with each other to form twisted strings. Another aspect that is possible to observe is that on day 3 the dead cells (red) were located inside the biofilm while the live cells were externalized, but on day 5 the predominance of dead cells occurred.

**Figure 5:**
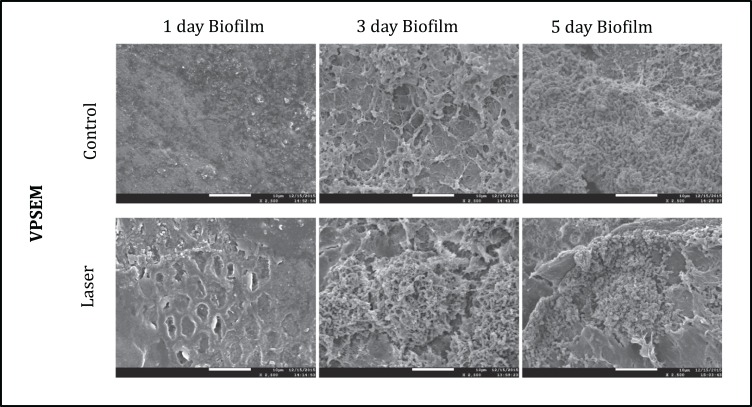
Morphology and structure of after one, three and five days *S. mutans* biofilms imaged by FESEM (2500X).

The SEM images of each group at different time points were shown in Fig. 5. The images illustrated the effects of enamel CO_2_ laser irradiation on the morphology and structure on *S. mutans* biofilm. On day 1, both groups had less bacteria in the biofilm than the day 3 and 5. Specimens with five days biofilm presented a thick and dense biofilm. Irradiated group had biofilms similar to those of composite control in both days. However, on day 1, the SEM observation revealed evidence of melting and fusion in the specimens treated with the CO_2_ laser.

## Discussion

Laser irradiation has long been used in medicine and lately also in the dental field. Some of these applications are intended for areas where bacteria are harbored or used directly to eradicate bacteria from infected areas (Sol et al., 2011). Another aspect to consider is that the critical pH (5.5) for the dissolution of enamel is reduced to 4.8 after irradiation with CO_2_ laser (Fox et al., 1992). Despite a variety of experiments demonstrating the inhibitory effect of CO_2_ laser in reducing enamel demineralization, little is known about the effect of surface irradiation on bacterial growth.

The wavelengths obtained with CO_2_ lasers (λ = 9.3, 9.6, 10.3 and 10.6 μM) produce radiation in the infrared region, which coincides with some absorption bands of hydroxyapatite, particularly the carbonate and phosphate groups (Rodrigues, dos Santos & Featherstone, 2006). When the light is absorbed in a few external micrometers from the surface of the tooth and converted the enamel, a heat loss occurs in mineral carbonate as well as fusion of the hydroxyapatite crystals, resulting in a decrease of acid reactivity in this structure (Fried et al., 1997). The reduced solubility of spent enamel has also been attributed to melting and recrystallization of the crystals (Nelson et al., 1986; Nelson et al., 1987). These morphological changes can interfere in the default adhesion of bacterial cells to the tooth surface, which is essential to early carious lesions formation (Newbrun, 1977).

Adhesion is the initial step in biofilm formation. Thus, an understanding of bacteria-surface interactions is essential for biofilm control. Bacterial cells approach surfaces by different means, including sedimentation, movement with liquid flow, bacterial motility with cell surface appendages, and interaction with other cells to form aggregates (Teughels et al., 2006). In this study, initial adhesion in day 1 of biofilm formation was investigated and the data revealed that laser irradiation decreased the initial cell adherence of *Streptococcus mutans*. On day 3, it is possible to observe a greater value of biofilm formation in control group. However, with the progression of time, at day 5, this difference becomes less visible and is statistically similar.

The formation and composition of biofilm appear to vary on different surfaces (Aroonsang et al., 2014) and effects of material/surface properties, such as surface charge, hydrophobicity, roughness, topography, and chemistry on bacterial adhesion and biofilm formation have been investigated for many years (Anselme et al., 2010; Badihi Hauslich et al., 2013; Guégan et al., 2014; Perera-Costa et al., 2014; Song & Ren, 2014). These factors may be interrelated, which may explain the inhibition of biofilm formation found on irradiated enamel.

The role of hydrophobicity in oral bacterial adhesion has been reviewed elsewhere (Busscher, Norde & van der Mei, 2008; Busscher et al., 2010; Nobbs, Lamont & Jenkinson, 2009). In general, by tuning the hydrophobicity of a surface, bacterial adhesion can be inhibited. The results of this present study indicated that laser irradiation was able to increase the hydrophobicity of the enamel when compared to the control group. This finding was in agreement with a previous report by Quirynen et al. (1996), who showed that in oral environments on supragingival surfaces, less biofilm is formed on hydrophobic surfaces than hydrophilic ones. This increase of the hydrophobicity on irradiated enamel can be related to the decrease in initial biofilm formation which was found in this study.

Gene expression in bacteria can be affected by light and laser irradiation (Steinberg et al., 2008). However, how bacteria sense and respond to different surface properties at the genetic level is largely unknown. *S. mutans* does not always dominate within dental plaque, but it is recognized that glucosyltransferases (Gtfs) from *S. mutans* play critical roles in the development of virulent dental plaque. These Gtf genes, among other functions, are responsible for producing the soluble and insoluble polysaccharides matrix. The EPS-insoluble plays a significant role on *S. mutans* adhesion and accumulation on the tooth surface (Bowen & Koo, 2011). In addition, it potentially changes the biofilm structure, resulting in increased porosity (Dibdin & Shellis, 1988), which allows fermentable substrates to diffuse and be metabolized in the deepest parts of the biofilm (Zero, van Houte & Russo, 1986). The present study demonstrated that irradiating enamel surface with laser irradiation did not affect the gene expression of GtfB and, consequently, did not change the production of polysaccharides. Conversely, synthesis of glucan binding proteins (Gbps) may enhance the ability of *S. mutans* to interact with the EPS-rich matrix (Banas & Vickerman, 2003). The adhesion between the bacterial cells and the EPS-matrix may be partially mediated by cell-surface GbpC, and possibly GbpB whereas secreted GbpA and GbpD may be cross-linked with the matrix contributing to the maintenance of the biofilm architecture (Lynch et al., 2007). The amounts of GbpB observed in irradiated enamel do not have direct implications for the biofilm morphogenesis and structural integrity (Duque et al., 2011).

To determine the morphology of *S. mutans* biofilms with respect to topography, we used SEM microscopy. In our study, the *S. mutans* biofilm topography was visibly not altered after laser irradiation, in both fluorescence microscopy and SEM images. This result suggests that although the laser irradiation promoted surfaces alteration such as fusion and melt, which is visible in MEV it did not promote disorganization and disaggregation of the microorganisms in the biofilm, inhibiting their growth and metabolism.

To the best of our knowledge, this is the first report of CO_2_ laser irradiation effect on the prevention of oral biofilm development. Laser irradiation modified *S. mutans* biofilm development by reducing its formation. Our findings suggest that bacteria have complex systems to sense and respond to environmental challenges. The interplay between how surface properties and pellicle formation affect the bacterial adhesion strength, the mechanical stability, and detachment of biofilms, is an area that needs to be elucidated. In conclusion, CO_2_ laser irradiation can modify the energy surface and disrupt the initial biofilm formation.

## Supplemental Information

10.7717/peerj.2458/supp-1 Supplemental Information 1 Contact Angle.

10.7717/peerj.2458/supp-2 Supplemental Information 2 1 day Biofilm.

10.7717/peerj.2458/supp-3 Supplemental Information 3 Polysaccharides.

10.7717/peerj.2458/supp-4 Supplemental Information 4 2 and 3-day Biofilm.

## Additional Information and Declarations

### Data Deposition

The following information was supplied regarding data availability: The raw data has been supplied as Supplemental Dataset Files.
