# Supplementary material for: Effects of CO2 laser irradiation on matrix-rich biofilm development formation–an in vitro study
Source: PeerJ. 2016 Nov 1;4:e2458. doi: 10.7717/peerj.2458 (PMC5101588; doi:10.7717/peerj.2458)
Supplement: Supplemental Information 1 [file peerj-04-2458-s001.pdf]

	Contact Angle	
	Laser	Control
	99,00	71,10
	92,40	75,00
	73,50	75,80
	86,10	78,80
	88,30	79,40
**Average**	87,86	76,02
**SD**	9,41	3,33
