# Supplementary material for: Effects of CO2 laser irradiation on matrix-rich biofilm development formation–an in vitro study
Source: PeerJ. 2016 Nov 1;4:e2458. doi: 10.7717/peerj.2458 (PMC5101588; doi:10.7717/peerj.2458)
Supplement: Supplemental Information 2 [file peerj-04-2458-s002.pdf]

	Log 10	
	Laser	Control
	5,875	6,342
	5,176	6,000
	5,653	6,267
	5,771	5,648
	5,914	6,169
	4,978	5,878
	5,618	5,937
		6,267
Average	5,569	6,064
SD	0,357	0,239
