# Supplementary material for: Effects of CO2 laser irradiation on matrix-rich biofilm development formation–an in vitro study
Source: PeerJ. 2016 Nov 1;4:e2458. doi: 10.7717/peerj.2458 (PMC5101588; doi:10.7717/peerj.2458)
Supplement: Supplemental Information 3 [file peerj-04-2458-s003.pdf]

	Insoluble Polysaccharides			
	Day 3		Day 5	
	Laser	Control	Laser	Control
	8,836	6,526	10,403	7,950
	8,154	6,873	8,563	8,154
	8,775	8,965	7,473	5,777
	13,061	8,134	9,858	13,402
	4,951	7,541	9,880	8,904
	5,428	5,156	7,405	
Average	8,20	7,20	8,93	8,84
DP	2,92	1,33	1,31	2,80

	Soluble Polysaccharides			
	Day 3		Day 5	
	Laser	Control	Laser	Control
	3,659	6,127	6,867	3,412
	4,975	5,469	3,906	4,153
	3,412	4,153	3,906	5,222
	7,114	3,741	3,824	9,005
	3,166	6,538	3,442	3,330
	3,577	3,495	3,906	4,235
Average	4,32	4,92	4,31	4,89
DP	1,51	1,29	1,27	2,13
