# Supplementary material for: Effects of CO2 laser irradiation on matrix-rich biofilm development formation–an in vitro study
Source: PeerJ. 2016 Nov 1;4:e2458. doi: 10.7717/peerj.2458 (PMC5101588; doi:10.7717/peerj.2458)
Supplement: Supplemental Information 4 [file peerj-04-2458-s004.pdf]

	Day 3					
	CFU / mL		Dry Weight (mg/mL)		CFU / Dry Weight	
	Laser	Control	Laser	Control	Laser	Control
	8,000	7,301	6,400	6,200	6,884	7,614
	7,477	7,544	6,900	7,200	7,030	7,546
	7,176	7,653	6,500	6,600	7,062	7,687
	7,875	7,699	7,700	6,400	7,109	7,738
	7,477	7,176	6,000	6,700	6,953	7,718
	7,813	7,845		6,200	6,921	7,870
	8,000	7,699				8,541
	7,699	8,176				7,725
	8,000	7,699				8,148
		8,000				
Average	7,724	7,679	6,700	6,550	6,993	7,843
DP	0,292	0,297	0,644	0,378	0,088	0,313

	Day 5					
	CFU / mL		Dry Weight (mg/mL)		CFU / Dry Weight	
	Laser	Control	Laser	Control	Laser	Control
	8,000	7,301	9,800	18,500	7,009	6,034
	7,477	7,544	9,900	10,400	6,481	6,527
	7,176	7,653	10,200	6,500	6,167	6,840
	7,875	7,699	12,200	3,300	6,789	7,180
	7,477	7,176	10,600	12,800	6,452	6,069
	7,813	7,845	9,800	9,700	6,822	6,860
	8,000	7,699		18,500	7,009	7,015
	7,699	8,176			6,613	6,682
	8,000	7,699			7,009	7,363
		8,000				7,180
Average	7,724	7,679	10,417	11,386	6,706	6,775
DP	0,292	0,297	0,926	5,720	0,298	0,455
